# Novel biomarkers for prediction of mortality after acute exacerbation of chronic obstructive pulmonary disease

**DOI:** 10.1186/cc12072

**Published:** 2013-03-19

**Authors:** H Michalopoulou, H Michalopoulou, P Stamatis, F Kattis, D Stamatis

**Affiliations:** 1 Metaxa Hospital, Athens, Greece

## Introduction

Retrospective studies suggest that cardiac troponin levels are often elevated in patients with acute exacerbation of chronic obstructive pulmonary disease (AECOPD) indicating a poor survival. Novel high-sensitivity cardiac troponin (hs-cTnT) assays have better analytical precision than standard troponin (cTnT) assays. We elaborated a prospective cohort study to investigate the prognostic value of this novel biomarker in patients with AECOPD.

## Methods

Fifty-six patients (mean age 64 years, 68% male) with the final diagnosis of AECOPD were enrolled. Those who were diagnosed with acute coronary syndromes were excluded. We measured cardiac troponin T with a standard fourth-generation assay and a high-sensitivity assay. Clinical, electrocardiographic and echocardiographic data were collected at admission and the primary prognostic endpoint was death during 30 days of follow-up.

## Results

Mean hs-cTnT levels at admission were 34 ng/l. During the follow-up period seven patients (12%) died. Thirty-eight percent of patients had hs-cTnT above the range of 14 ng/l. Prognostic accuracy of hs-cTnT for death was significantly higher, with area under the ROC curve (AUC) of 0.83 (95% CI: 0.72 to 0.86), than that of cTnT (AUC: 0.63, 95% CI: 0.56 to 0.74; *P <*0.001). After adjustment for age, gender, creatinine levels, heart rate, left ventricular ejection fraction, arterial O_2 _pressure and systolic pulmonary artery pressure, hs-cTnT above the 99th percentile was associated with a hazard ratio for death of 3.2 (95% CI: 1.5 to 6.7).

## Conclusion

In AECOPD, novel biomarkers such as hs-cTnT appear to be positively associated with COPD severity and could be a determinant of mortality.

